# Cloning, expression, purification and crystallization of a pair of novel virulence factors, SghA and SghR, from *Agrobacterium tumefaciens*

**DOI:** 10.1107/S2053230X15012881

**Published:** 2015-08-25

**Authors:** Fuzhou Ye, Chao Wang, Qinqin Fu, Lian-hui Zhang, Yong-gui Gao

**Affiliations:** a School of Biological Sciences, Nanyang Technological University, 60 Nanyang Drive, Singapore 637551, Singapore; b Institute of Molecular and Cell Biology, 61 Biopolis Drive, Singapore 138673, Singapore; c Division of Cellular and Molecular Research, National Cancer Centre Singapore, 11 Hospital Drive, Singapore 169610, Singapore

**Keywords:** SghA, SghR, crystallization, virulence factor, *Agrobacterium*, plant–microbe interaction, salicylic acid, lacI, chemical signal, glucosidase

## Abstract

Two proteins, SghA and SghR, which were recently identified and characterized as novel bacterial virulence factors regulating the infection of plant hosts by *Agrobacterium*, were cloned, overexpressed and purified with high yield. Both SghA and SghR form dimers in solution. The purified SghA and SghR were crystallized and the crystals diffracted to 1.9 and 2.1 Å resolution, respectively. Data were collected and processed, and the crystallographic parameters were within acceptable ranges. These results will help in the determination of their structures in order to uncover the molecular mechanism of how these two proteins together control the release of plant defence signals against agrobacteria during pathogen–host interaction.

## Abstract 2

The crystallization of the novel virulence factors SghA and SghR is reported.

## 1. Introduction   

The mechanisms of infection of their hosts by bacteria have been widely reported. However, how bacteria strategically control the biosynthesis of the infection machinery and the timely shutting off of the energy-consuming infection process once infection has been successfully established are largely unknown. *Agrobacterium tumefaciens* has been reported to be the causative agent of crown gall disease (the formation of plant tumours) in over 140 plant species, making it of great concern to the agricultural industry (Moore *et al.*, 1997 ▸). Agrobacteria start infection *via* integrating the oncogenic T-DNA (transferred DNA) from the bacterial tumour-inducing (Ti) plasmid into the genome of plant hosts, which also renders this bacterial pathogen a powerful tool for plant genetic modification (Gelvin, 2003 ▸; Tzfira & Citovsky, 2006 ▸). In our research, *A. tumefaciens* was chosen as an experimental model to decipher host–pathogen interaction. Infection by *A. tumefaciens* involves a number of virulence factors and concomitant plant-derived chemical signals such as salicylic acid (SA), indole-3-acetic acid (IAA) and quorum-sensing (QS) signal (Baron & Zambryski, 1995 ▸; Chevrot *et al.*, 2006 ▸; Yuan *et al.*, 2007 ▸; Zhang *et al.*, 2002 ▸; Liu & Nester, 2006 ▸). It has been reported that these signals play different roles in fine-tuning the responses of plants and bacteria at different time points during infection (Klessig *et al.*, 2000 ▸; Subramoni *et al.*, 2014 ▸).

SA is a well characterized plant defence signal in response to different pathogens (Delaney *et al.*, 1994 ▸; Gaffney *et al.*, 1993 ▸; Zottini *et al.*, 2007 ▸; Anand *et al.*, 2008 ▸). During *Agrobacterium* infection, SA can inhibit the expression of the *vir* genes and bacterial growth (Yuan *et al.*, 2007 ▸; Gohlke & Deeken, 2014 ▸). Unlike other plant-derived chemical signals (such as IAA and cytokinin), whose biosynthetic genes are carried in T-DNA (thus, the expression of T-DNA promotes the *de novo* synthesis of IAA and cytokinin in plant hosts), no genes are found in T-DNA for the biosynthesis of SA (Akiyoshi *et al.*, 1987 ▸; Hwang *et al.*, 2010 ▸; Liu *et al.*, 1982 ▸). In contrast, the timely release of SA takes place by enzymatic hydrolysis of its storage conjugated form SA 2-*O*-β-d-glucoside (SAG). Evidence has demonstrated that the concentration of SA is low at the beginning of bacterial infection, but dramatically increases along with a stronger and stronger systematically acquired resistance at the late stage of *Agrobacterium* infection (Albert, 2013 ▸; Gohlke & Deeken, 2014 ▸; Lee *et al.*, 2009 ▸). The accumulation of endogenous SA at the late stage is in turn closely correlated with T-DNA integration and *vir* gene expression (Albert, 2013 ▸; Ditt *et al.*, 2001 ▸; Veena *et al.*, 2003 ▸). However, how the timely regulation of the SA level during the bacterial infection takes place remains elusive.

Our unpublished results identified that a pair of novel proteins, SghA and SghR, from *A. tumefaciens* A6 are responsible for the temporal regulation of SA concentration in plants during *Agrobacterium* infection, which is independent of the typical VirA/VirG signalling pathway (Stachel & Zambryski, 1986 ▸). Sequence analyses revealed that SghA belongs to glucosidase hydrolase family 1. A *BLAST* search of the PDB found several homologue structures, the top two among which are BcBgl from *Bacillus circulans* subsp. *alkalophilus* (PDB entry 1qox; Hakulinen *et al.*, 2000 ▸) and Tmbgl from *Thermotoga maritima* (PDB entry 1od0; Zechel *et al.*, 2003 ▸). The sequence identities of these two proteins to SghA are 45.0 and 43.0%, respectively. Our functional studies demonstrate that SghA specifically hydrolyzes the inactive SAG to release the active SA, which subsequently inhibits the expression of other *vir* genes (VirA, VirD2, VirE2 *etc.*), ultimately avoiding the energy-consuming process of biosynthesizing the infection machinery. This strategy helps *Agrobacterium* colonization and saves energy for spreading the infection in a self-controlled mode. Furthermore, we identified a transcription factor SghR in *A. tumefaciens* A6, a homologue of Atu1522 from *A. tumefaciens* C58, that negatively regulates the transcription of *sghA* at an early stage of bacterial infection *via* physically binding to its promoter region. SghR assembles as a member of the lacI family of transcription factors containing an N-terminal DNA-binding domain and a C-terminal regulatory domain (Bell & Lewis, 2000 ▸; Lewis *et al.*, 1996 ▸; Lewis, 2005 ▸). Sequence alignment of SghR with the reported lacI family member Atu1522 (PDB entry 3gv0; New York SGX Research Center for Structural Genomics, unpublished work) from *A. fabrum* strain C58 and Cagg_2268 (PDB entry 3bbl; New York SGX Research Center for Structural Genomics. unpublished work) gave 91.2 and 25.7% identity, respectively. However, the Atu1522 structure did not contain the N-terminal DNA-binding domain. Experiments have indicated that both SghA and SghR control tumour growth during *Agrobacterium* infection and SghA plays a role in the late stage when the infection has been successfully established. Here, we report our preliminary data, including cloning, expression, purification, crystallization and data collection, on these two novel virulence factors.

## 2. Materials and methods   

### 2.1. Cloning, expression and purification of SghA and SghR   

Genes encoding SghA and SghR from *A. tumefaciens* A6 were amplified by PCR using the primers 5′-CCGCTCGAGATGGATGACGAAAGGGC-3′ (forward) and 5′-CCG­CTCGAGAAAGCCTCACCCCTTC-3′ (reverse) for SghA and 5′-CCGCTCGAGATGAACGATACTGGTA­ATTCCG-3′ (forward) and 5′-CCGCTCGAGGCGTTCCTTCTATCA­AGG-3′ (reverse) for SghR using *A. tumefaciens* A6 genomic DNA as the PCR template. Detailed molecular cloning information for SghA and SghR is listed in Tables 1 ▸ and 2 ▸, respectively. The amplified fragments were inserted into the expression vector pET-14b. The recombinant plasmids were verified by DNA sequencing and then transformed into *Escherichia coli* BL21 CodonPlus(DE3) RIL cells for protein expression.

For large-scale expression of SghA protein, the *E. coli* BL21 cells were cultured in 2×YT medium with antibiotics (100 µg ml^−1^ ampicillin and 34 µg ml^−1^ chloramphenicol) at 37°C. When the optical density (OD_600_) of the cell cultures reached ∼0.8, protein expression was induced by adding 0.5 m*M* isopropyl β-d-1-thiogalactopyranoside (IPTG) at 16°C. After 18 h of induction, the cells were harvested by centrifugation (5000 rev min^−1^, 30 min, 4°C). The cell pellets were resuspended in lysis buffer [50 m*M* Tris–HCl pH 7.5, 150 m*M* NaCl, 20 m*M* imidazole, 5 m*M* β-mercaptoethanol (β-ME)]. The cell suspension was lysed with a Panda disruptor (GEA Niro Soavi, Italy) and clarified by centrifugation (22 000 rev min^−1^, 20 min, 4°C). The supernatant was collected and filtered through a 0.45 µm Minisart filter unit (Sartorius Biotech). Subsequently, the filtered supernatant was loaded onto a 5 ml Ni–NTA column (GE Healthcare) and eluted with a linear gradient increase of imidazole concentration (0.02–0.5 *M*). After SDS–PAGE analysis, fractions containing the target proteins were pooled together and dialyzed against 50 m*M* Tris–HCl pH 7.5, 50 m*M* NaCl, 5 m*M* β-ME). Samples were further purified by anion-exchange chromatography with a HiTrap Q HP Column (GE Healthcare). Elution was conducted with a linear gradient of NaCl concentration (0.05–1 *M*) and analyzed by SDS–PAGE. The target proteins were then pooled together, concentrated and loaded onto a HiLoad Superdex 200 26/60 gel-filtration column pre-equilibrated with buffer consisting of 50 m*M* Tris–HCl pH 7.5, 50 m*M* NaCl, 1 m*M* tris(2-carboxyethyl)phos­phine (TCEP). After column elution and checking by SDS–PAGE, the target proteins were collected, concentrated to 17 mg ml^−1^, flash-frozen in liquid nitrogen and stored at −80°C.

For SghR protein preparation, the bacterial cells were harvested using a protocol similar to that for SghA. The cells were resuspended and lysed in 50 m*M* NaH_2_PO_4_ pH 8.5, 300 m*M* NaCl, 5 m*M* β-ME and clarified by centrifugation (22 000 rev min^−1^, 30 min, 4°C). The sample was first loaded onto an Ni–NTA affinity column (GE Healthcare) and then eluted with a linear gradient increase of imidazole concentration (0–0.5 *M*). After checking by SDS–PAGE, the fractions containing the target protein were pooled, concentrated and loaded onto a HiLoad Supderdex 200 26/60 gel-filtration column pre-equilibrated with 50 m*M* HEPES pH 7.0, 50 m*M* NaCl, 2 m*M* TCEP. The target protein was eluted, concentrated to 6.4 mg ml^−1^, flash-frozen in liquid nitrogen and stored at −80°C for subsequent experiments.

### 2.2. Molecular-weight calibration of SghA and SghR   

To calculate the molecular weights of SghA and SghR in solution, the High Molecular Weight (HMW) gel-filtration calibration kit (GE Healthcare) was used. The gel-filtration column (Superdex 200, 10/300 GL) was first equilibrated with the sample buffer (50 m*M* HEPES pH 7.0, 50 m*M* NaCl, 2 m*M* TCEP). Blue dextran 2000 (to determine the void volume), a mixture of four standard proteins (ovalbumin, 44 000 Da; conalbumin, 75 000 Da; aldolase 2, 158 000 Da; ferritin 2, 440 000 Da), SghA and SghR were sequentially loaded onto the column in four separate runs. Following the instructions for the kit, the *K*_av_ was plotted against log(molecular weight). Consequently, the molecular weights of SghA and SghR in solution were determined.

### 2.3. Crystallization and data collection   

Crystallization screening of SghA (diluted to 4 mg ml^−1^) and SghR (at 6.4 mg ml^−1^) was performed. For each condition, three varied ratios of protein and reservoir solution (0.2:0.1 µl, 0.15:0.15 µl and 0.1:0.2 µl) were screened by the sitting-drop vapour-diffusion method using a Phoenix robot (Art Robbins Instruments) at 20°C. Screening kits from Hampton Research, including Crystal Screen, Crystal Screen 2, Index, PEG/Ion, PEGRx and SaltRx, were used. Detailed information on SghA and SghR crystallization is listed in Table 3 ▸.

For SghA, initial crystals were obtained in a condition consisting of 20%(*w*/*v*) PEG 3350, 0.4 *M* ammonium formate using 0.1 µl protein solution and 0.1 µl reservoir solution. Further optimization by fine-tuning the PEG concentration (19–21%) and increasing the drop size to 1 µl protein solution and 1 µl reservoir solution was carried out applying both the hanging-drop and the sitting-drop methods. Only the hanging-drop method gave decent crystals. We did not obtain any crystals by the sitting-drop method as most wells precipitated. After the crystals reached maximum size (10 d), they were cryoprotected in crystallization buffer containing 35% PEG 3350 before flash-cooling in liquid nitrogen.

For SghR, initial crystal hits were observed in a condition consisting of 0.05 *M* HEPES Na pH 7.0, 20% PEG 3350, 1%(*w*/*v*) tryptone using 0.2 µl protein solution and 0.1 µl reservoir solution. As the tryptone in the crystallization condition is a bacterial nutrient, the drop is easily contaminated by bacteria, which interfere with the crystallization process. To avoid bacterial contamination, all reagents used for SghR optimization were filtered through a 0.2 µl filter and the optimization plates were set up in a fume cupboard. The crystallization condition was optimized by varying the PEG concentration (17–22%) and increasing the drop size to 2 µl protein solution and 1 µl reservoir solution. The crystals were cryoprotected with a cryoprotectant consisting of 35% PEG 3350 in the reservoir and were then flash-cooled in liquid nitrogen.

Diffraction-quality data sets for both SghA and SghR were collected at 100 K on beamline I04 at Diamond Light Source (DLS) and the data were processed using *XDS* (Kabsch, 2010 ▸). Detailed information on data collection is given in Table 4 ▸.

## 3. Results and discussion   

**Figure 1:**
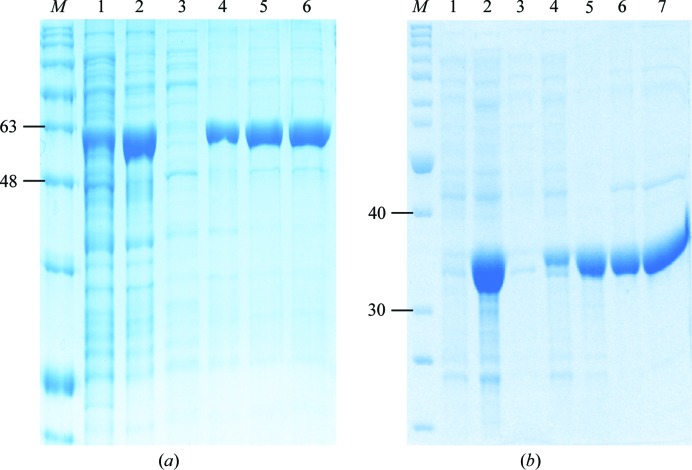
Expression and purification of SghA (*a*) and SghR (*b*). (*a*) Lane *M*, molecular-weight marker (labelled in kDa); lanes 1 and 2, supernatant and pellet after cell extraction, respectively; lane 3, flowthrough after Ni–NTA affinity binding; lanes 4 and 5, after elution from Ni–NTA affinity column; lane 6, purified SghA protein after gel-filtration chromatography. (*b*) Lane *M*, molecular-weight marker (labelled in kDa); lanes 1 and 2, total cells before and after IPTG induction, respectively; lane 3, flowthrough after Ni–NTA affinity binding; lanes 4 and 5, supernatant and pellet after cell extraction, respectively; lane 6, after elution from Ni–NTA purification; lane 7, purified SghR after gel-filtration chromatography.

**Figure 2:**
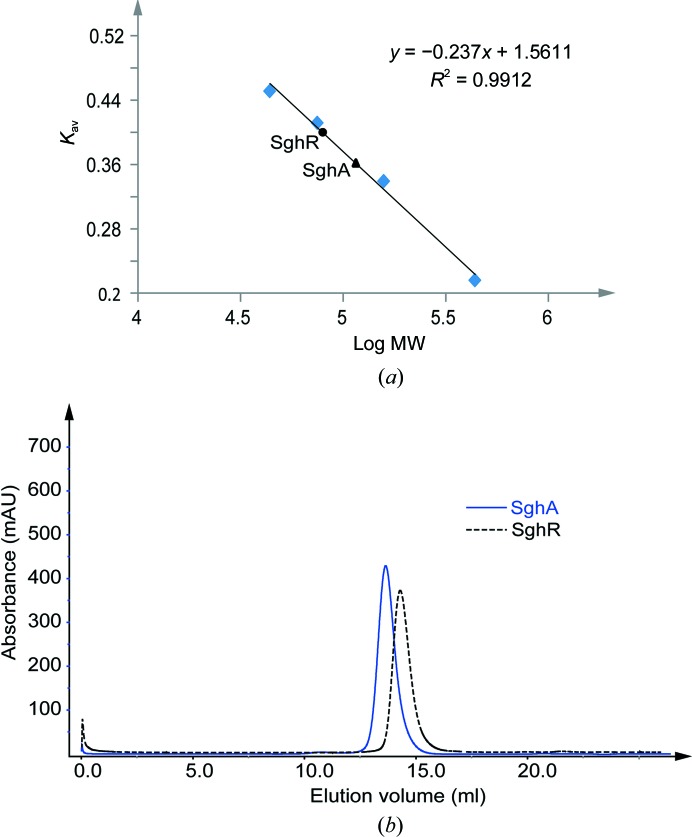
(*a*) Standard calibration curve based on the log(molecular weight) of four different standard proteins plotted against *K*_av_. The circle and triangle on the standard curve indicate the positions of SghR and SghA, respectively (depicted based on the *K*_av_ value). (*b*) Gel-filtration chromatography elution profile of SghA (solid line) and SghR (dashed line) used for molecular-weight calibration.

The recombinant plasmids pET-14b-SghA and pET-14b-SghR encode the corresponding target proteins with an N-terminal His_6_ tag. Purification of SghA included three chromatographic steps (sequentially, Ni^2+^-affinity, anion-exchange and gel-filtration chromatography). In contrast to SghA, two chromatographic steps were adopted for the purification of SghR: Ni^2+^-affinity and gel-filtration chromatography. The purified SghA and SghR proteins both displayed high purity (Figs. 1 ▸ *a* and 1 ▸ *b*), with a final yield of approximately 50 mg protein per litre of culture. It was noted that there was only a subtle difference in lane 6 in Fig. 1 ▸(*a*) and lane 7 in Fig. 1 ▸(*b*) from the corresponding nickel-affinity purification. This result suggested that the proteins were quite pure after nickel-affinity purification and that the subsequent anion-exchange or gel-filtration chromatography was not essentially able to enhance the protein purity. From the gel-filtration chromatography profiles, the size of both proteins also appeared to be double that of their corresponding monomers. To verify this, the molecular weights of SghA and SghR were calibrated using a HiLoad Superdex 200 10/300 gel-filtration column and the High Molecular Weight calibration kit (GE Healthcare). The calibration results demonstrated that both proteins indeed exist as dimers in solution (Fig. 2 ▸).

**Figure 3:**
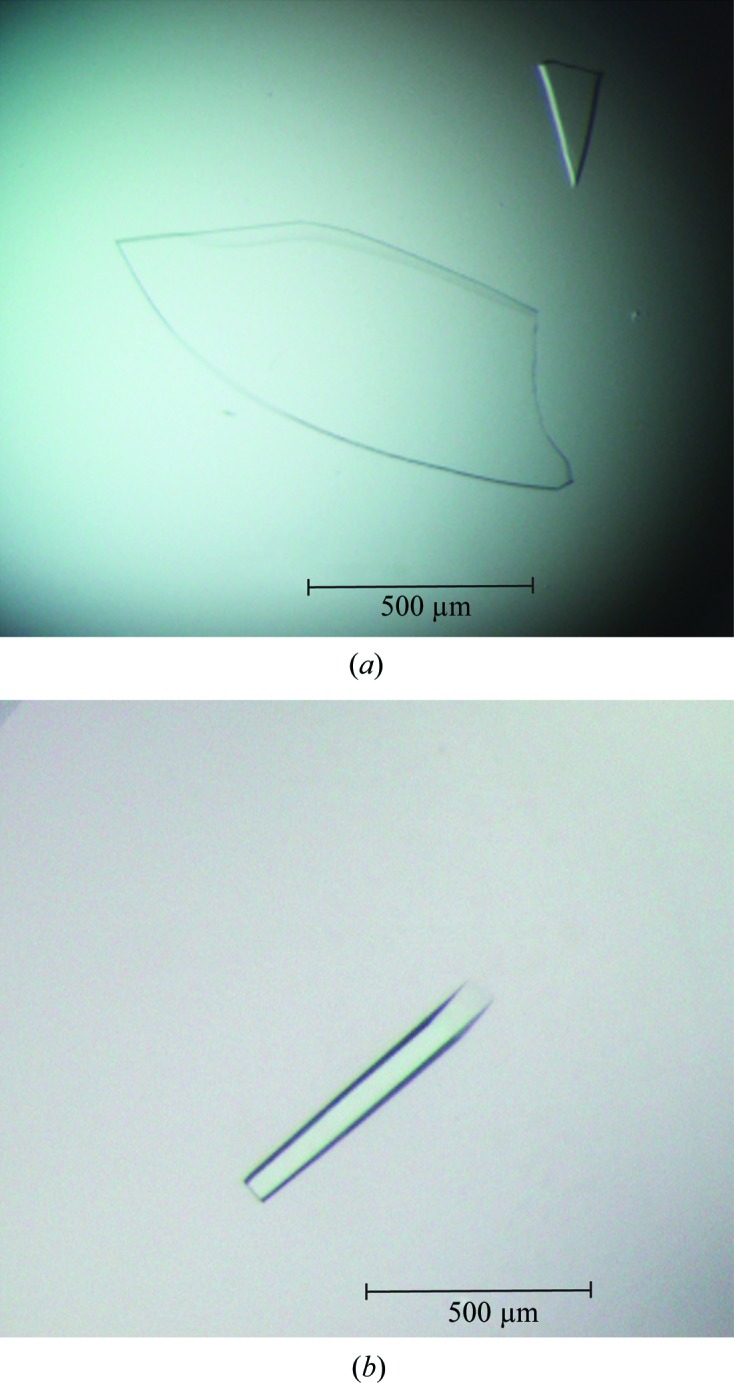
Crystal photographs of SghA (*a*) and SghR (*b*).

**Figure 4:**
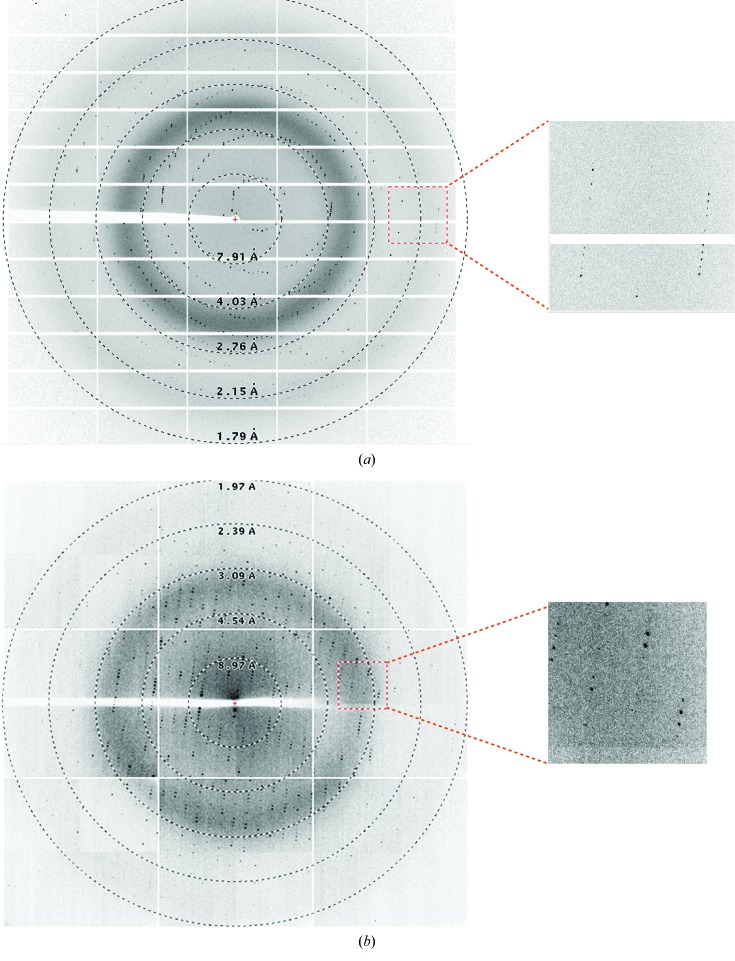
Diffraction patterns of SghA (*a*) and SghR (*b*). The right panel is a magnification of the boxed region. The resolution rings were generated using *ADXV* (http://www.scripps.edu/~arvai/adxv.html).

Crystallization screening and further optimization led to decent plate-shaped and rod-shaped crystals of SghA and SghR, respectively (Fig. 3 ▸). Both crystals were harvested with thorough washing with their reservoir solutions. Subsequent SDS–PAGE separation and mass-spectral identification were performed, and the results clearly indicated the crystallized macromolecules were the target proteins SghA and SghR, respectively. Therefore, data collection was carried out and the collected data sets were processed to acceptable *R*_merge_, completeness and 〈*I*/σ(*I*)〉 values in the highest resolution bin (Fig. 4 ▸ and Table 4 ▸). Both the SghA and the SghR crystals belonged to space group *P*2_1_2_1_2_1_. The data from the SghA crystals were processed to 1.9 Å resolution, with unit-cell parameters *a* = 64.2, *b* = 80.4, *c* = 184.62 Å, whereas the data from the SghR crystals were processed to 2.1 Å resolution, with unit-cell parameters *a* = 35.54, *b* = 119.45, *c* = 121.94 Å (Table 1 ▸). *V*_M_ calculations indicated that there are two molecules in the asymmetric unit. On the basis of our molecular-weight calibration results, it appears that the crystals of both proteins contained a dimer in the asymmetric unit. Further model building and structure refinement are ongoing for both structures.

SghA was identified as a novel virulence factor that shuts off the infection machinery through controlling the release of active SA from SAG to inhibit the expression of the other *vir* genes after infection has been established successfully. Its enzyme specificity toward SAG and its catalytic mechanism will be further investigated by structural study based on the current crystallographic data and on substrate/product-bound forms of SghA together with other enzymatic assays. As a transcriptional repressor, SghR represses the transcription of *sghA via* physically binding to its promoter region and de­repressing the transcription upon sensing the environmental stimulus. Structural insights into the regulation mechanism of SghA by SghR through the determination of SghR–effector and SghR–DNA complexes are also under way.

## Figures and Tables

**Table 1:** Macromolecule-production information for SghA

Source organism	*A. tumefaciens*
DNA source	Genomic DNA
Forward primer†	CCGCTCGAGATGGATGACGAAAGGGC
Reverse primer†	CCGCTCGAGAAAGCCTCACCCCTTC
Cloning vector	pET-14b
Expression vector	pET-14b
Expression host	*E. coli* BL21 CodonPlus(DE3) RIL
Complete amino-acid sequence of the construct produced‡	MGSSHHHHHHSSGLVPRGSHMLEMDDERAYPMTDHKALAARFPGDFLFGVATASFQIEGATKVDGRKPSIWDAFCNMPGHVFGRHNGDVACDHYNRWEDDLDLIKEMGVEAYRFSIAWPRIIPDGFGPINEKGLDFYDRLVDGCKARGIKTYATLYHWDLPLTLMGDGGWASRSTAHAFQRYAKTVMARLGDRLDAVATFNEPWCAVWLSHLYGIHAPGERNMEAALAAMHHINLAHGFGVEASRHVAPKVPVGLVLNAHSVIPASNSDADMKAAERAFQFHNGAFFDPVFKGEYPAEMIEALGSRMPVVEAEDLSIISQKLDWWGLNYYTPMRVADDATEGAEFPATKQAPAVSDVKTDIGWEVYAPALHSLVETLYERYELPDCYITENGACYNMGVENGEVDDQPRLDYYAEHLGIVADLVKDGYPMRGYFAWSLMDNFEWAEGYRMRFGLVHVDYETQVRTLKNSGKWYSALASGFPKGNHGVMKG

† XhoI restriction sites are underlined.

‡ The extra amino acids introduced into the wild-type SghA protein by cloning are underlined. The primary sequence of the SghA protein listed here corresponds to that reported by Henkel *et al.* (2014 ▸).

**Table 2:** Macromolecule-production information for SghR

Source organism	*A. tumefaciens*
DNA source	Genomic DNA
Forward primer†	CCGCTCGAGATGAACGATACTGGTAATTCCG
Reverse primer†	CCGCTCGAGGCGTTCCTTCTATCAAGG
Cloning vector	pET-14b
Expression vector	pET-14b
Expression host	*E. coli* BL21 CodonPlus(DE3) RIL
Complete amino-acid sequence of the construct produced‡	MGSSHHHHHHSSGLVPRGSHMNDTGNSGRDEAKATTGERPTLKTIAYMTGLGITTVSRALKDAPDIGAETKERVRLIAQQIGYQPNRAGVRLRTGKTNVIALVLSVDEELMGFTSQMVFGITEVLATTQYHLVVTPHTHAKDSMVPIRYILETGSADGVIISKIEPNDPRVRFMTERKMPFVTHGRSDMGIEHAYHDFDNEAYAYEAVERLAQCGRKRIAIIVPPSRFAFHDHARKGFTRGIRDFGVSEFPLDAITIETPLDKIRDFGKRLMQSDDRPDGIVSISGSSTIALVAGFEAAGVRIGKDIDIVSKQSAEFLNWIQPQIHTVNEDIKLAGRELAKALLARINGAPPETLQSVSRPVWSSMAPKP

† XhoI restriction sites are underlined.

‡ The extra amino acids introduced into the wild-type SghR protein by cloning are underlined. The primary sequence of the SghA protein listed here corresponds to that reported by Henkel *et al.* (2014 ▸).

**Table 3:** Crystallization of SghA and SghR

Protein	SghA	SghR
Method	Vapour diffusion	Vapour diffusion
Plate type	24-well hanging drop	24-well hanging drop
Temperature (K)	293	293
Protein concentration (mgml^1^)	4	6.4
Buffer composition of protein solution	50m*M* TrisHCl pH 7.5, 50m*M* NaCl, 1m*M* TCEP	50m*M* HEPES pH 7.0, 50m*M* NaCl, 2m*M* TCEP
Composition of reservoir solution	20%(*w*/*v*) PEG 3350, 0.4*M* ammonium formate	0.05*M* HEPES Na pH 7.0, 20% PEG 3350, 1%(*w*/*v*) tryptone
Volume and ratio of drop†	2l, 1:1	3l, 2:1
Volume of reservoir (l)	400	400

† The volume ratio is that of protein:reservoir.

**Table 4:** Data-collection and processing statistics Values in parentheses are for the outer shell.

Data set	SghA	SghR
Diffraction source	I04, DLS	I04, DLS
Wavelength ()	0.9795	0.9795
Temperature (K)	100	100
Crystal-to-detector distance (mm)	245	287.7
Rotation range per image ()	0.2	0.4
Total rotation range ()	90	90
Exposure time per image (s)	1	1
Space group	*P*2_1_2_1_2_1_	*P*2_1_2_1_2_1_
Unit-cell parameters (, )	*a* = 64.2, *b* = 80.4, *c* = 184.62, = = = 90	*a* = 35.54, *b* = 119.45, *c* = 121.94, = = = 90
Average mosaicity ()	0.26	0.32
Resolution range ()	501.9 (2.101.90)	502.1 (2.212.10)
Total No. of reflections	263299	110508
No. of unique reflections	73481	31202
Completeness (%)	99.7 (100)	99.7 (99.9)
Multiplicity	3.4 (3.4)	3.5 (3.6)
*I*/(*I*)	8.8 (2.5)	9.6 (3.7)
*R*_merge_ (%)	10.0 (47.1)	8 (32.7)
Overall *B* factor from Wilson plot (^2^)	18.5	23.1
